# Chemometric Analysis for Identification of Botanical Raw Materials for Pharmaceutical Use: A Case Study Using *Panax notoginseng*

**DOI:** 10.1371/journal.pone.0087462

**Published:** 2014-01-31

**Authors:** Jieqiang Zhu, Xiaohui Fan, Yiyu Cheng, Rajiv Agarwal, Christine M. V. Moore, Shaw T. Chen, Weida Tong

**Affiliations:** 1 Pharmaceutical Informatics Institute, College of Pharmaceutical Sciences, Zhejiang University, Hangzhou, China; 2 Office of New Drug Quality Assessment, Center for Drug Evaluation and Research (CDER), US Food and Drug Administration, Silver Spring, Maryland, United States of America; 3 Office of New Drugs, Center for Drug Evaluation and Research (CDER), US Food and Drug Administration, Silver Spring, Maryland, United States of America; 4 National Center for Toxicological Research (NCTR), US Food and Drug Administration, Jefferson, Arkansas, United States of America; University of Bonn, Institut of experimental hematology and transfusion medicine, Germany

## Abstract

The overall control of the quality of botanical drugs starts from the botanical raw material, continues through preparation of the botanical drug substance and culminates with the botanical drug product. Chromatographic and spectroscopic fingerprinting has been widely used as a tool for the quality control of herbal/botanical medicines. However, discussions are still on-going on whether a single technique provides adequate information to control the quality of botanical drugs. In this study, high performance liquid chromatography (HPLC), ultra performance liquid chromatography (UPLC), capillary electrophoresis (CE) and near infrared spectroscopy (NIR) were used to generate fingerprints of different plant parts of *Panax notoginseng*. The power of these chromatographic and spectroscopic techniques to evaluate the identity of botanical raw materials were further compared and investigated in light of the capability to distinguishing different parts of *Panax notoginseng*. Principal component analysis (PCA) and clustering results showed that samples were classified better when UPLC- and HPLC-based fingerprints were employed, which suggested that UPLC- and HPLC-based fingerprinting are superior to CE- and NIR-based fingerprinting. The UPLC- and HPLC- based fingerprinting with PCA were able to correctly distinguish between samples sourced from rhizomes and main root. Using chemometrics and its ability to distinguish between different plant parts could be a powerful tool to help assure the identity and quality of the botanical raw materials and to support the safety and efficacy of the botanical drug products.

## Introduction

In recent years, there has been increased interest in the United States in developing botanical preparations as pharmaceutical products and not only as dietary supplements. Since it is known that different plant parts of a herbal medicine may possess different treatment effects, one hurdle has been to develop analytical methods to adequately identify the source, i.e., different plant parts, of the botanical raw material to ensure that the botanical drug substance and drug product can be reproducibly manufactured to provide the same safety and efficacy as the clinical trial supplies,. A typical example for dramatic differences in therapeutic activity is *Ephedrae herba* and *Ephedrae Radix et Rhizoma*. *Ephedrae herba* is the herbaceous stem part of Ephedra which can elevate blood pressure and *Ephedrae Radix et Rhizoma* is the root part, which can lower blood pressure [1]. In order to avoiding medication errors with herbal preparations, regulatory agencies, such as the US FDA [2], EMA [3]and China SFDA [4], recommend that herbal medicines are prepared from specific parts of the botanical raw material.

There are many reports about fingerprint techniques to address the identity and quality of botanicals, which are mainly chromatographic analysis, including high performance liquid chromatography (HPLC) [5], [6], gas chromatography (GC) [7], ultra performance liquid chromatography (UPLC) [8], [9] and capillary electrophoresis (CE) [10]. Spectroscopy methods are also applied to gain fingerprints. Near infrared spectroscopy [11] is a widely used technology in the pharmaceutical industry, which has advantages such as real-time measurement. These methods can be compared in order to determine their advantages and drawbacks and to provide assurance on how to obtain meaningful chromatographic fingerprints to identify the quality of botanical drug products. Furthermore, in combination with chemometric approaches, fingerprint technology can be applied as a powerful method for characterizing botanical drug of different origins and quality. For example, pattern recognition methods, such as principal component analysis (PCA), hierarchical cluster analysis(HCA), linear discriminant analysis (LDA), k-nearest neighbor (k-NN), soft independent modeling of class analogy(SIMCA), partial least squares-discrimination analysis (PLS-DA) are commonly applied for distinguishing different origins of botanical drugs.

In this study, *Panax notoginseng (Burk.) F.H. Chen* (Also named as Tianqi or Sanqi in China) was used for analysis. Not only is it an important Chinese herbal medicine which has a diversity of effects, including anticarcinogenic [12], hepatoprotective [13] and cardiovascular protective properties [14], [15], but the different plant parts are used for different therapeutic purposes. In China, the rhizome and the main root of *Panax notoginseng* are supplied separately in the market, with the rhizome parts extracted for “XUESAITONG” while the main root is used for “XUESHUANTONG”.

**Figure 1:**
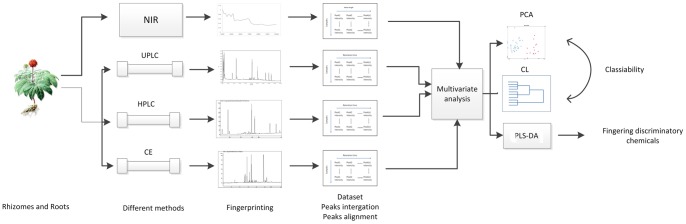
Workflow for the study design.

In this study, three chromatographic fingerprinting methods and one spectroscopic fingerprinting method were developed using high performance liquid chromatography (HPLC), ultra performance liquid chromatography (UPLC), capillary electrophoresis (CE), and near infrared spectroscopy (NIR). As illustrated in the workflow of study design shown in Fig.1, their power for distinguishing different parts of *Panax notoginseng* using chemoinformatics approaches were compared and investigated.

## Materials and Methods

### Materials and Reagents

HPLC grade acetonitrile was purchased from Merck (Darmstadt, Germany). Acetic acid glacial was obtained from Tedia (Fairfield, OH, USA). Distilled water was purified by Milli-Q system (Millipore, USA). Ginsenosides Rg1, Re, Rb1, Rd1 and notoginsenoside R1 were purchased from Jilin University (Changchun, China). The other chemicals were of analytical grade.

### Plant Material

In total, 45 batches of dried *Panax notoginseng* samples were studied to build a model, which consists of 16 batches of rhizomes, and 29 batches of main roots. 6 additional batches of samples were used to test and validate the model. The main root parts of the botanical raw material *Panax notoginseng* were collected from Yunnan and Guangxi Province, and the rhizomes are collected from Yunan Province, China. The plant materials were collected within one year and used as commercial products. The botanical origin of materials was identified morphologically by Gan Pingyuan (Wenshan Institute for Drug Control, Yunnan Province, China) and Zhu Jieqiang (Zhejiang University).

### Ethics

No specific permissions were required for the described field studies. The locations are neither privately owned nor protected by the Chinese government. No endangered or protected species were sampled.

### Sample Preparation

The *Panax notoginseng* sample was pulverized and passed through a 280 µm screen. 40 ml of 70% methanol (v/v) was added to 0.5 g powdered sample. The operating parameters were optimized according to reference [8] for high efficacy of extracting saponins. The suspension was extracted by an ultrasonicator (40 kHz, Shumei KQ250-E, Shanghai, China) for 60 min. During the sonication process, the temperature was controlled below 60°C. After cooling, the extracts were filtered and the filtrate was evaporated to dryness *in vacuo*. The residue was transferred into a 5 ml volumetric flask and diluted to the desired volume with 70% methanol. The solution was filtered through a 0.22 µm nylon membrane (ANPEL, Shanghai, China) before analysis.

### HPLC Fingerprints of *Panax notoginseng*

The HPLC method conditions were optimized to get a robust separation, including columns, mobile phase, temperature and gradient. The HPLC system used was an Agilent 1100 instrument (Agilent Technologies, USA) which consisted of a quaternary solvent delivery system, an auto-sampler, an on-line degasser, a column temperature controller and ultraviolet detector. The chromatographic separation was performed using an Agilent Zorbax Eclipse Plus C18 column (4.6×50 mm i.d.; 1.8 µm particle size) (Agilent, USA). Flow rate was 0.8 ml/min and the detection wavelength was 203 nm. The column temperature was set at 35°C and the injection volume was 3 µl. The mobile phases consisted of water (solvent A) and acetonitrile (solvent B). The elution conditions were: 0–22 min, 17–19% B; 22–30 min, 19–27% B; 30–35 min, 73% B; 35–47 min, 27–46% B; 47–70 min, 46–90% B. The re-equilibrium was 15 min; the total run time was 85 min.

### UPLC Fingerprints of *Panax notoginseng*

UPLC method was employed from [16]. UPLC was performed on a Waters ACQUITY UPLCTM system, equipped with a binary solvent delivery system and an auto sampler. Chromatographic separation was carried out on an ACQUITY UPLCTM CSH C18 column (2.1×50 mm i.d.; 1.7 µm particle size) (Waters Co., MA, USA). The mobile phase consisted of water-formic acid (A; 100∶0.01, v/v) and acetonitrile-acetic acid (B; 100∶0.01, v/v). The gradient elution was as follows: 19–20% B at 0–6 min; 20–31% B at 6–8.5 min; 31–33% B at 8.5–11 min; 33–90% B at 11–17 min; 90% B at 17–19 min, and a 10 min re-equilibrium was conducted before the next injection. The column was maintained at 45°C with the flow rate of 0.35 ml/min. The detection wavelength was set at 203 nm. The injection volume was 5 µl.

### CE Fingerprints of *Panax notoginseng*

The capillary electrophoresis method was according to the method as described [17], with some parameter adjustment. In this study, an HP3D capillary electrophoresis system (Agilent, Waldbronn, Germany) equipped with diode-array detector was used. Capillary electrophoresis was performed on a 80.0 cm (71.5 cm to the detector) ×75 µm I.D. fused silica capillary (Polymicro Technologies, USA). The detection wavelength was 195 nm and the temperature was 25°C. The separation voltage was controlled at −27 kV. The running buffer solution was prepared by mixing 5.0 ml 280 mM SDS, 1.0 ml 200 mM H_3_PO_4_ in water, 2.0 ml acetonitrile and 1.5 ml 2-propanol in a 10 ml volumetric flask and dilute with water to volume. All solutions were filtered through a 0.22 µm nylon membrane. The injection mode was pressure injection, 50 mbar for 10 seconds.

### HPLC-MS^n^ Analysis

Analysis was performed on an Agilent 1100 series LC system equipped with a Finnigan LCQ Deca XP^plus^ ion trap mass spectrometer (Thermo Finnigan, USA) via an ESI interface. The chromatographic conditions were the same as the HPLC fingerprint method. The tune method for MS were as follows: collision gas, ultra high purity helium (He); nebulizing gas, high purity nitrogen (N_2_); the source voltage for positive and negative mode were 4.0 kV and −3.0 kV, respectively; sheath gas (N_2_) at a flow rate of 60 arbitrary units; auxiliary gas (N_2_) at a flow rate of 10 arbitrary units; capillary temperature, 350°C; capillary voltage for positive and negative mode were 19 V and −15 V, respectively. The collision energy for MS^n^ spectra was 30%.

### NIR Analysis

An Antaris MX FT-NIR spectrophotometer (Thermo-Fisher Co., Madison, USA) equipped with integrating sphere was used to collect the NIR spectra. According to the reported method with slight adaption [11]. The wave number range is 4000–10,000 cm^−1^. Each spectrum was measured with 4 cm^−1^ data interval and obtained by averaging 64 times.

### Chromatographic Method Validation

Five main chemicals (notoginsenoside R1, ginsenoside Re, ginsenoside Rg1, ginsenoside Rb1 and ginsenoside Rd) were selected as markers for chromatographic method validation. The instrument precision was tested by six consecutive injections of a sample solution; the RSD was below 3%. The inter-day precision was determined by six replicate measurements of a sample, the RSD was less than 3%. The samples were stable for 24 h.

### Data Analysis

All the chromatographic peaks were integrated and aligned according to our laboratory standard practice [18]. Firstly, the chromatographic peaks were integrated. Then, the results were introduced into Similarity Evaluation System for Chromatographic Fingerprint of Traditional Chinese Medicine (Version 2004A, National Committee of Pharmacopoeia, China). After aligning all the peaks, the reference chromatogram was generated by reserving peaks above 0.1% of the area percent. Profiles containing 53, 39 and 28 peaks were selected from UPLC, HPLC and CE, respectively (Detailed in Figure S1). The NIR spectra were pretreated with moving average and 1st derivative. The resulting data was imported to ArrayTrack software 3.4.5 (NCTR, USA) for cluster analysis. The MATLAB was used to perform PCA analysis. The SIMCA-P software 11.0 (Umetrics, Sweden) was used to perform PLS-DA analysis.

## Results and Discussion

**Figure 2:**
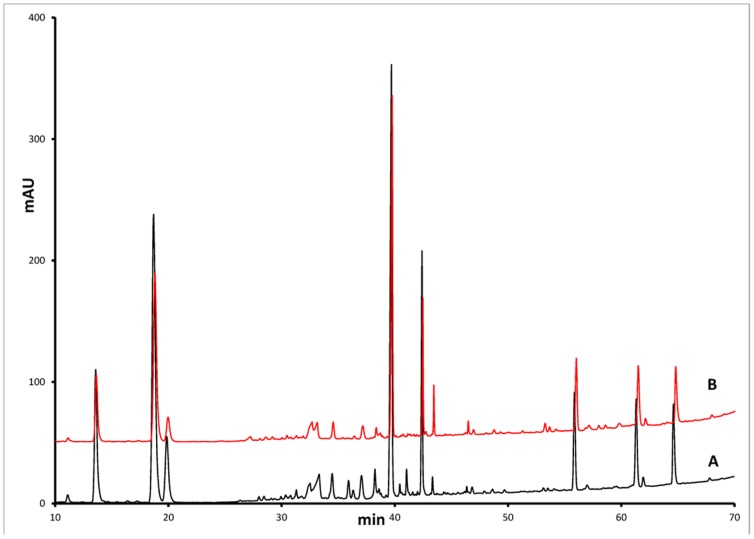
Typical HPLC chromatograms. (A) rhizomes and (B) main roots.

The traditional method of characterization is through comparison of HPLC spectra. As shown in Fig.2, for the plant parts for *Panax notoginseng*, the HPLC fingerprints appear to be very similar. However, since these spectra are highly complex and contain many classes of compounds, the comparison is often highly qualitative which can lead to missed features or unnecessarily tight requirements. We believe that use of chemometric techniques to analyze the spectra would provide a higher level of assurance that important characteristics are not overlooked, and provide consistency in the final botanical drug products. A similar approach has been successfully applied to the complex naturally-derived molecule of heparin to provide classification of pure and impure heparin [19], as well as quantification of heparin impurities [20].

### Chromatographic Fingerprints of *Panax notoginseng*

**Figure 3:**
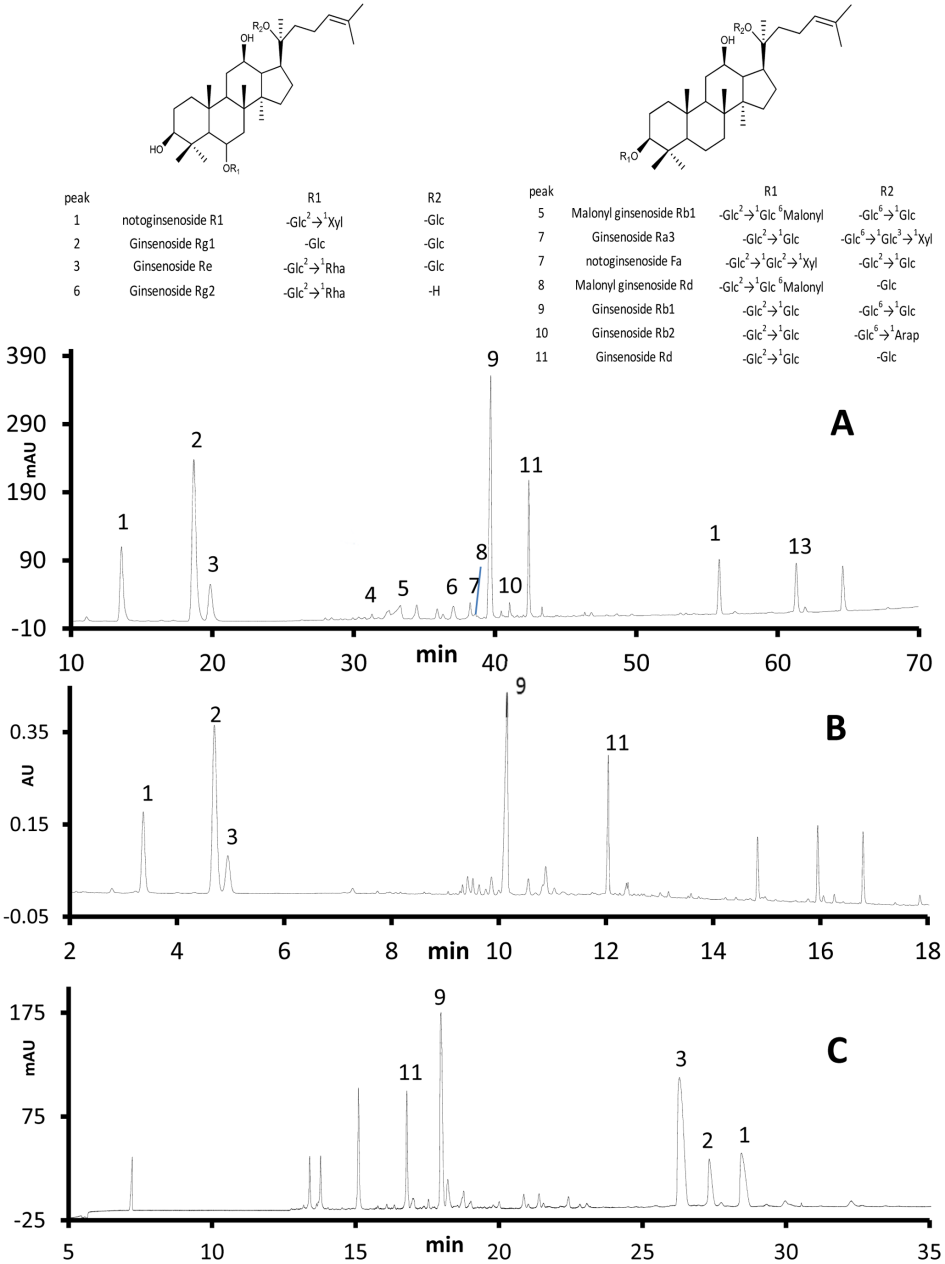
Typical chromatograms of rhizome of P. notoginseng. (a) HPLC-UV, (b) UPLC-PDA and (c) CE-UV, including (1) notoginsenoside R1,(2) ginsenoside Rg1, (3) ginsenoside Re, (9) ginsenoside Rb1, (11) ginsenoside Rd.

**Figure 4:**
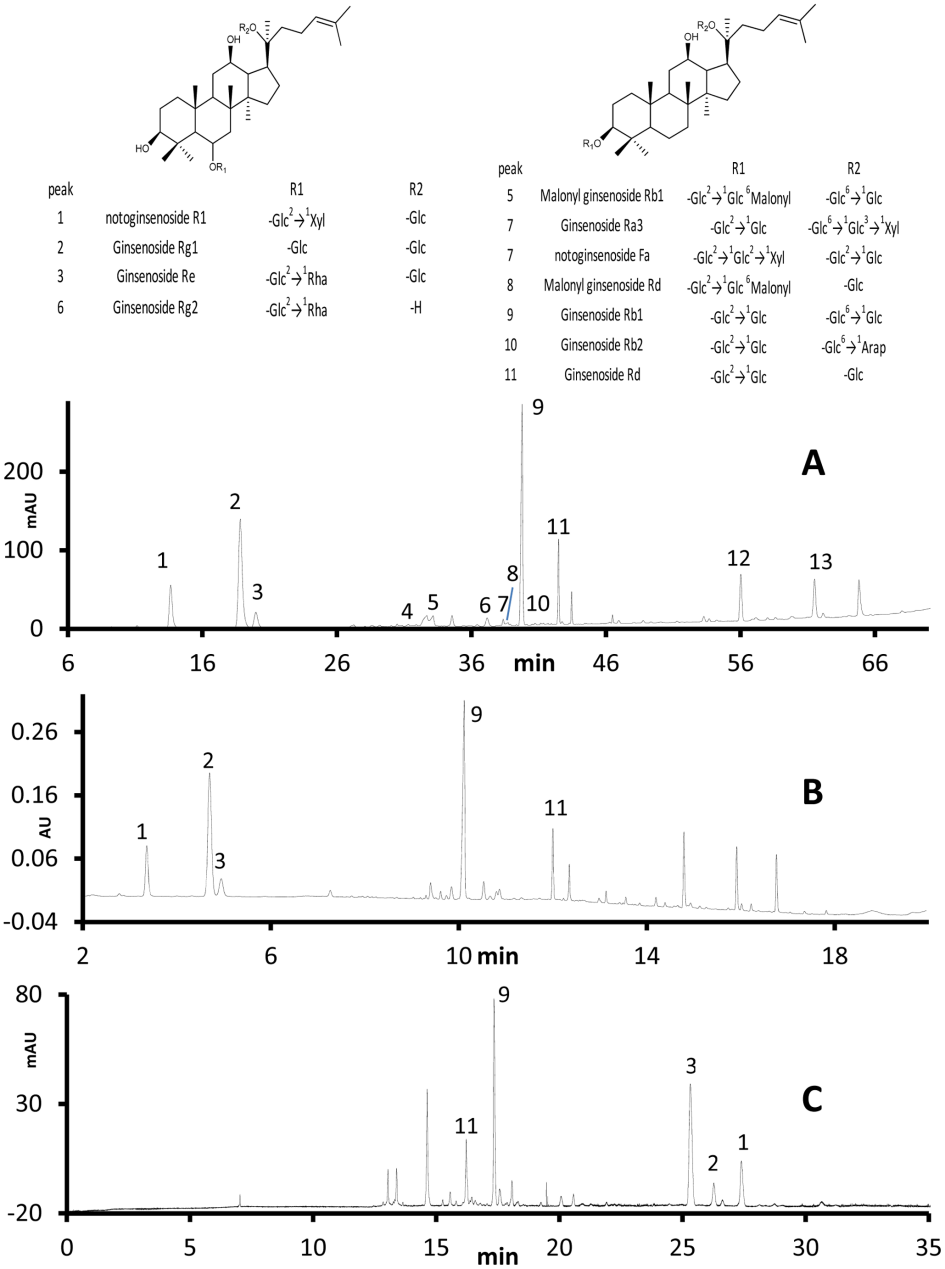
Typical chromatograms of main roots of P. notoginseng. (A) HPLC-UV, (B) UPLC-PDA and (C) CE-UV.

The typical chromatograms generated for rhizomes and main roots from UPLC, HPLC and CE are shown in Fig.3 and Fig.4, respectively. UPLC has a number of advantages over the other chromatographic methods. UPLC utilized the least run time among the three methods. Due to its higher peak capacity and greater resolution, it identified the most chemical information while the analysis time is only 1/3 of analysis time of HPLC, and 1/2 of the analysis time of CE. UPLC also separated more components from the mixture than the other techniques, coming closest to the earlier published reports [21], [22]. To date over 50 saponins in *Panax notoginseng* [23] have been identified, which occur in small amounts and vary widely. The UPLC has a higher column efficiency as a result of advancements in the particle size which has made it possible to distinguish small peaks from the baseline noise. Another advantage of UPLC was its reduction in the consumption of mobile phase, which is more friendly to the environment and more economical. Due to the smaller size of packing particles in column, the samples need more carefully pretreating for UPLC methods.

### Principal Component Analysis

In botanical drug studies, PCA is a commonly-used multivariate tool for classification and discrimination [24]. It is an unsupervised clustering technique for reducing the dimensionality of a data set, without losing important information. The PCA analysis is formulated as Eq (1):
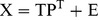
(1)


**Figure 5:**
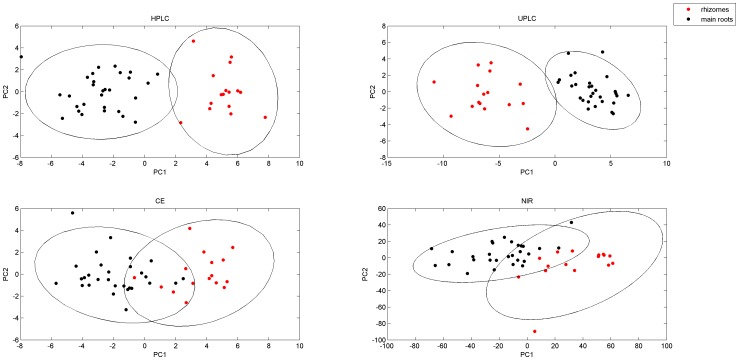
PCA scores plots of four methods with 95% confidence ellipses. red dot are rhizomes, black dot are main roots.

Where X is the data matrix, consists of m rows of samples and n columns of peaks. T is the score vector matrix. P*^T^* is the loading matrix and E is the residuals. The pre-treat method was auto-scale. The PCA scores plots of the three data sets are shown in Fig.5. (The loading plots are available in Figure S2). The first two PCs of all method accounted for over 50% of variability, represent a good summary of data variability. The ellipses are 95% confidence limits of each subclass. The PCA scores plot of HPLC and UPLC represent the least overlap of ellipse, clear separation of rhizomes and main roots was observed. In contrast, the ellipses in the score plot of NIR and CE have a larger overlap. The CE fingerprint based PCA plot was not satisfactory, in which 1 rhizome sample is misclassified as main root and 2 main root samples are located between rhizome samples. The NIR fingerprint-based PCA plot can distinguish rhizome and main root, but not as clearly as HPLC and UPLC. These results clearly suggest that UPLC-based and HPLC-based fingerprinting provides better discriminating ability than CE-based fingerprinting for the *Panax notoginseng* preparation. The boundary space between rhizomes and main roots in the NIR-based fingerprinting is winding and cramped, but on the whole it is a viable choice if adequately validated for the plant under consideration and if the other methods are not available.

### Cluster Analysis

**Figure 6:**
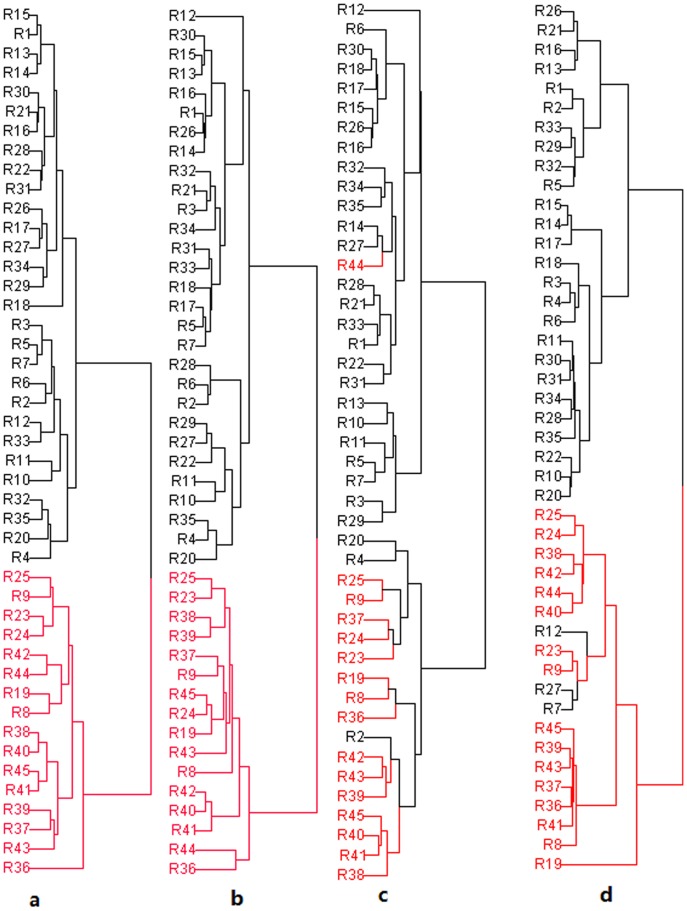
Dendrograms resulting of cluster analysis. (A) UPLC data, (B) HPLC data (C) CE and (D) NIR data. The red colored samples represent the rhizomes.

Hierarchical Cluster Analysis (HCA) is an unsupervised pattern recognition method for clustering samples based on the similarities between samples [25]. The hierarchical clustering was performed by ArrayTrack. The pre-treat method was auto scale. A method called dual cluster was applied, with Euclidean distance and Ward’s linkage type used [26]. The dendrograms are shown in Fig.6. The classification results were similar to the PCA analysis. The HPLC and UPLC-based fingerprints correctly classified all the samples. The CE-based fingerprints misclassified 3 main root samples. The NIR-based fingerprints misclassified 3 main root as rhizomes, indicating that different chemometrics models lead to different discriminant results.

### Challenging the PCA and HCA Model

**Figure 7:**
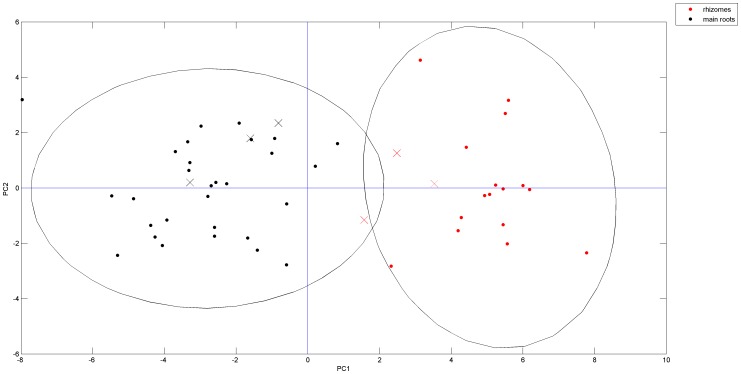
PCA scores plots of the testing samples. The red cross represent the testing rhizome samples. The black cross are mainroot samples.

**Figure 8:**
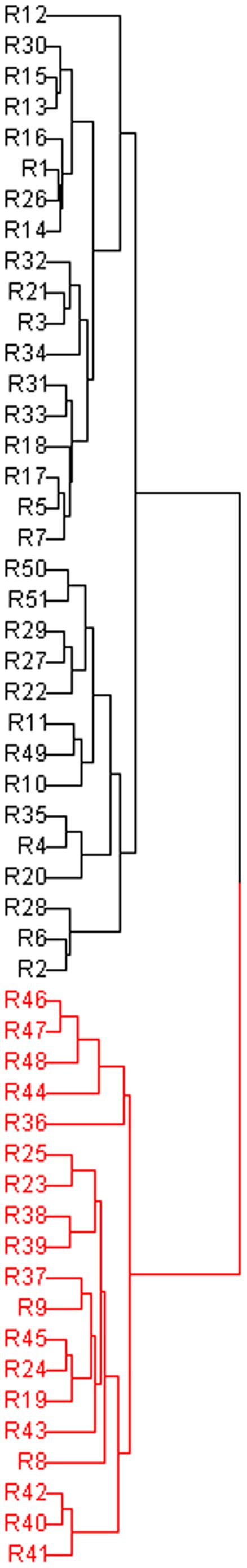
Dendrograms resulting of testing samples.

Six additional samples, which consisted of 3 rhizomes and 3 main roots, were used to test the established discriminant models. As mentioned above, HPLC methods clearly distinguished the rhizomes and main roots. And it is the most available method in the lab. The testing procedure was carried out by HPLC methods. The results are shown in figure 7 and 8. The 6 additional testing samples were correctly assigned to their own classes, indicating the applicability of the model for practical use.

### PLS-DA Analysis

In order to find the chemical differences between different parts of *Panax notoginseng*, a PLS-DA model was applied on SIMICA-P 11.0 software using the HPLC dataset. The discriminatory variables were sought out by the variable importance projection (VIP) value. The variables with larger VIP values were regarded as more relevant for classification. Variables whose VIP values were more than 1.14 are listed in Table 1. As previously mentioned, “XUESAITONG” and “XUESHUANTONG”, are two botanical drugs made from different parts of *Panax notogiseng*. Using the correct parts of raw materials is very important for guaranteeing the preparation’s quality. These components with larger VIP values may be used as quality markers for discriminating different parts of *Panax notoginseng* in practice.

**Table 1:** The discriminatory variables identified from VIP values.

Peak(figure3.a)	Retention time(min)	VIP value	Possible identification
5	33.26	1.496	Malonyl ginsenoside Rb1
10	40.97	1.435	Ginsenoside Rb2
4^a^	31.24	1.433	–
11	42.34	1.335	Ginsenoside Rd
12^a^	55.80	1.290	–
2	18.63	1.250	Ginsenoside Rg1
6	37.00	1.236	Ginsenoside Rg2
3	19.80	1.234	Ginsenoside Re
7	38.18	1.230	Ginsenoside Ra3/notoginsenoside Fa
13^a^	61.26	1.157	–
8	38.55	1.155	Malonyl ginsenoside Rd
9	39.64	1.144	Ginsenoside Rb1

a unkonw compound.

### Identification of Characteristic Peaks

Tentative identification of 9 of 12 characteristics peaks was accomplished by comparing against the ESI-MS^n^ data and retention times of standard saponins. The results are shown in Table 2.

**Table 2:** The identification of peaks in rhizome and main roots by LC-MS^n^.

Peak	RT(min)	[*M*-H]^−^	[*M*+Na]^+^	Identification	MS data (*m/z*)
2	18.63	799.8	823.5	Ginsenoside Rg1	637[M-H-Glc]^−^,619[M-H-H_2_O-Glc]^−^,475Agl
3	19.80	945.9	969.6	Ginsenoside Re	783[M-H-Glc]^−^,637[M-H-Glc-Rha]^−^,475Agl
4^b^	31.24	–	–	–	–
5^a^	33.26	1193.4	1217.6	Malonyl ginsenoside Rb1*	–
6	37.00	783.5	807.5	Ginsenoside Rg2	637[M-H-Rha]^−^,621[M-H-Glc]^−^,475Agl
7	38.18	1239.5	1263.6	Ginsenoside Ra3/notoginsenoside Fa	1107[M-H-Xyl]^−^,1077[M-H-Glc]^−^,945[M-H-Xyl-Glc]^−^,783[M-H-Xyl-2Glc]^−^
8^a^	38.55	1031.5	1055.6	Malonyl ginsenoside Rd*	–
9	39.64	1107.9	1131.6	Ginsenoside Rb1	945[M-H-Glc]^−^,783[M-H-2Glc]^−^,621[M-H-3Glc]^−^,459Agl
10	40.97	1077.9	1101.6	Ginsenoside Rb2	945[M-H-Arap]^−^,915[M-H-Glc]^−^,783[M-H-Arap-Glc]^−^,621[M-H-Arap-2Glc]^−^,459Agl
11	42.34	945.9	969.7	Ginsenoside Rd	783[M-H-Glc]^−^,621[M-H-2Glc]^−^,459Agl
12^b^	55.80	–	–	–	–
13^b^	61.26	–	–	–	–

a tentatively identified by [M-H]^−^.

b the peak purity was not good or mass signal was not clear.

Note: Xyl, xylose; Glc, glucose; Rha, rhamnose; Agl, aglycone; Arap, arabinose.

## Conclusion

Comparison of a number of analytical methods led to development of two optimized chromatographic and spectroscopic profiling methods, used with conventional multivariate analysis, to demonstrate the ability to distinguish between the rhizomes and main roots of the model species, *Panax notoginseng*. In a regulatory setting, having a simple methodology to ensure the identity of the raw material will help to ensure the quality of botanical drug products, providing evidence that approved products will provide similar safety and efficacy as the clinical trial supplies. In the future, these techniques could be used not only for control of approved products, but also to monitor the quality and identity of other herbal preparations, such as dietary supplements, which available in the marketplace and are not under the same rigorous control as approved pharmaceuticals. The power of these techniques could be used to preserve and protect the public health.

## Supporting Information

Figure S1 **The reference chromatograms of HPLC, UPLC and CE.** Showing all the peaks.
(TIF)

Figure S2 **The PCA loading plots of HPLC, UPLC and CE method.**
(TIF)
