# Supplementary figures and images for: Chemometric Analysis for Identification of Botanical Raw Materials for Pharmaceutical Use: A Case Study Using Panax notoginseng
Source: PLoS One. 2014 Jan 31;9(1):e87462. doi: 10.1371/journal.pone.0087462 (PMC3909187; doi:10.1371/journal.pone.0087462)

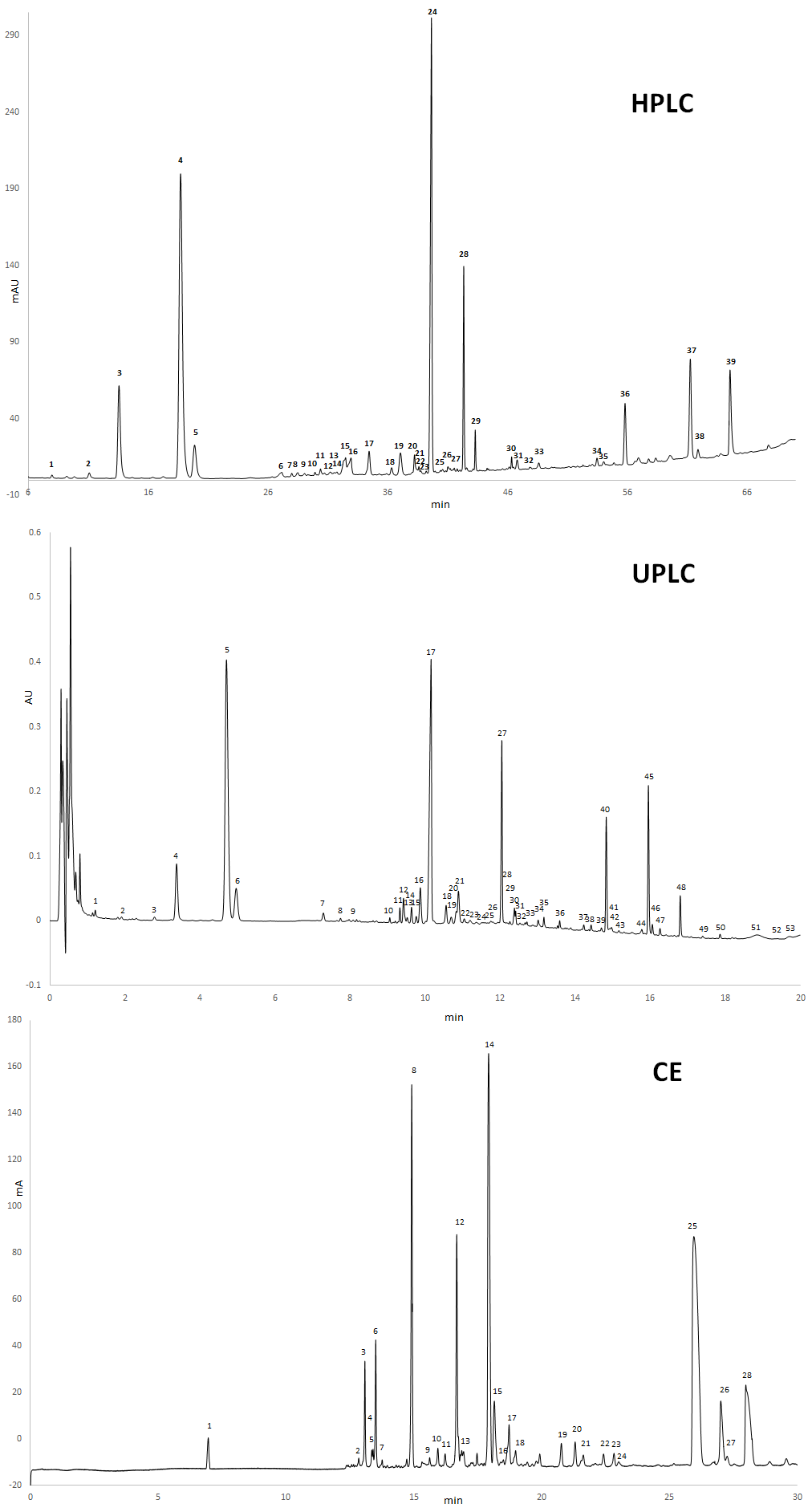

Supplement: Figure S1 — The reference chromatograms of HPLC, UPLC and CE. Showing all the peaks. (TIF) [file pone.0087462.s001.tif]

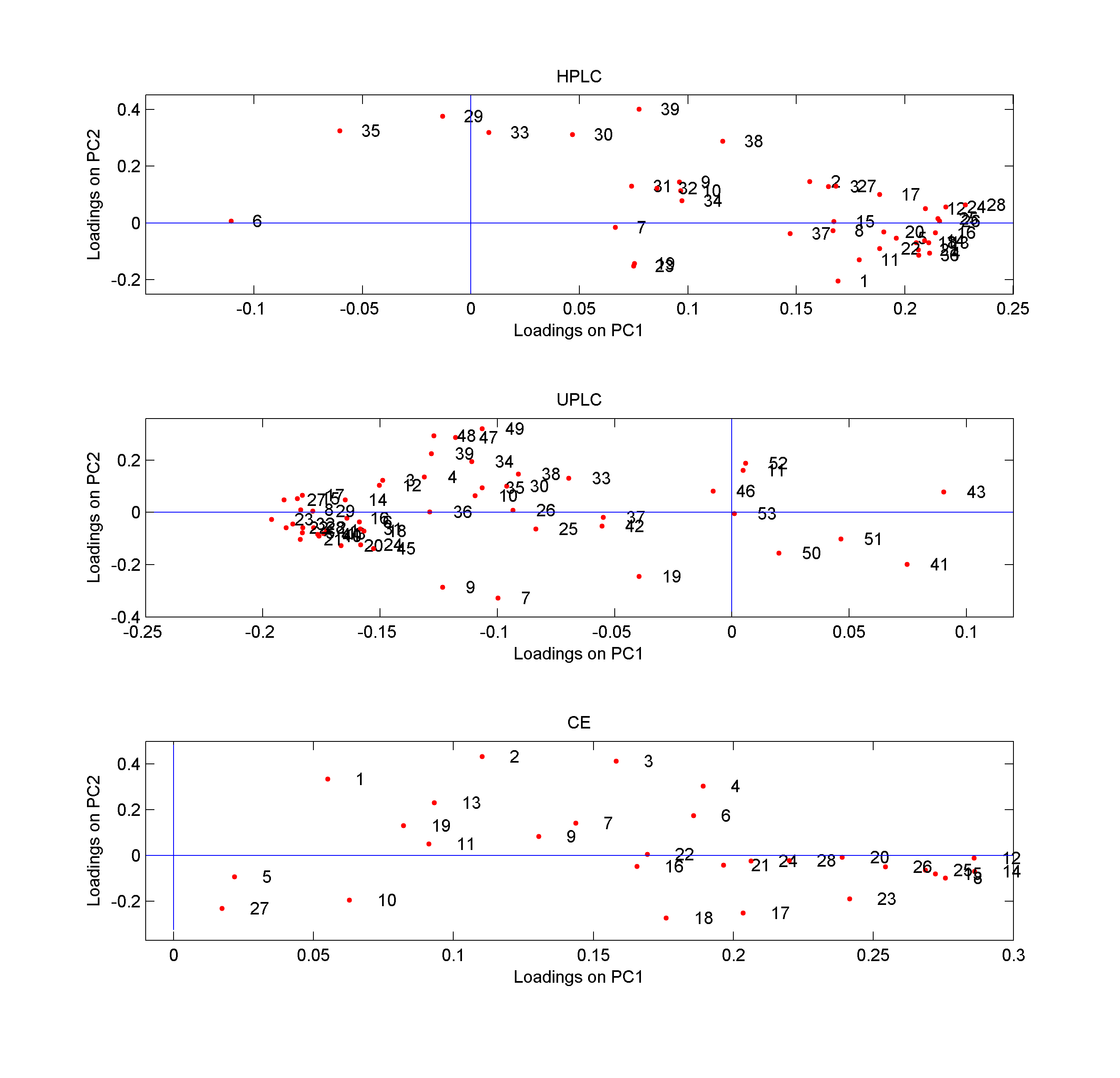

Supplement: Figure S2 — The PCA loading plots of HPLC, UPLC and CE method. (TIF) [file pone.0087462.s002.tif]
